# A randomized controlled phase III study of VB-111 combined with bevacizumab vs bevacizumab monotherapy in patients with recurrent glioblastoma (GLOBE)

**DOI:** 10.1093/neuonc/noz232

**Published:** 2019-12-07

**Authors:** Timothy F Cloughesy, Andrew Brenner, John F de Groot, Nicholas A Butowski, Leor Zach, Jian L Campian, Benjamin M Ellingson, Laurence S Freedman, Yael C Cohen, Noa Lowenton-Spier, Tamar Rachmilewitz Minei, Shifra Fain Shmueli, GLOBE Study Investigators, Nicholas Avgeropoulos, Joseph Beck, Tara Benkers, Felix Bokstein, Andrew Brenner, Eric Burton, Nicholas Butowski, Jian Campian, Jose Carrillo, Timothy Cloughesy, John de Groot, Paula De Robles, Jan Drappatz, Irine Dunbar, Karen Fink, Morris Groves, Xiaosi Han, Hormigo Adila, Randy Jensen, Agnieszka Kowalska, Pyriya Kumthekar, Mijung Lee, Glenn Lesser, Alexander Lossos, Rimas Lukas, David Macdonald, Aaron Mammoser, Laszlo Mechtler, Nimish Mohile, Seema Nagpal, Garth Nicholas, Teri Kreisl, Edward Pan, Scott Peak, Michael Pearlman, James Perry, Richard Peterson, David Piccioni, Henry Robins, Lara Ronan, Michael Salacz, David Schiff, David Tran, Leor Zach, Tzahala Tzuk-Shina, Tobias Walbert, Patrick Wen, Shlomit Youst, Patrick Y Wen

**Affiliations:** 1 Department of Neurology, David Geffen School of Medicine, University of California Los Angeles, Los Angeles, California, USA; 2 University of Texas Health San Antonio Cancer Center, San Antonio, Texas, USA; 3 Department of Neuro-Oncology, The University of Texas MD Anderson Cancer Center, Houston, Texas, USA; 4 Department of Neurological Surgery, University of California San Francisco, San Francisco, California, USA; 5 Oncology Institute, Chaim Sheba Medical Center, Tel HaShomer, Israel; 6 Division of Medical Oncology, Washington University School of Medicine, St Louis, Missouri, USA; 7 UCLA Brain Tumor Imaging Laboratory, Center for Computer Vision and Imaging Biomarkers, Department of Radiological Sciences, David Geffen School of Medicine, University of California Los Angeles, Los Angeles, California, USA; 8 Biostatistics and Biomathematics Unit, Gertner Institute for Epidemiology and Health Policy Research, Chaim Sheba Medical Center, Tel HaShomer, Israel; 9 VBL Therapeutics, Modi’in, Israel; 10 Center for Neuro-Oncology, Dana-Farber Cancer Institute, Boston, Massachusetts, USA; 11 University of Florida, Orlando Health, Orlando, FL; 12 Highlands Oncology Group, Rogers, Arizona; 13 Swedish Medical Center, Seattle, WA; 14 Tel Aviv Sourasky Medical Center, Tel Aviv, Israel; 15 University of Texas Health Science Center San Antonio, San Antonio, TX; 16 University of Louisville Physician, Louisville, KY; 17 University of California, San Francisco, San Francisco, CA; 18 Washington University School of Medicine, St. Louis, MO; 19 University of California Irvine Medical Center, Orange, CA; 20 University of California Los Angeles, Los Angeles, CA; 21 Anderson Cancer Center, Houston, TX; 22 Tom Baker Cancer Centre, Calgary, Alberta; 23 Hillman Cancer Center, Pittsburgh, PA; 24 Piedmont Physicians Neuro-Oncology, Atlanta, Georgia; 25 Neuro-Oncology Associates, Baylor University, Dallas, TX; 26 Texas Oncology-Austin Midtown, Austin, TX; 27 University of Alabama at Birmingham, Birmingham, AL; 28 Derald H. Ruttenberg Treatment Center, New York, NY; 29 Huntsman Cancer Institute at The University of Utah, Salt Lake City, UT; 30 Neurology Associates of Stony Brook, Stoney Brook, NY; 31 Northwestern University, Chicago, IL; 32 SUNY Upstate Medical Center, Syracuse, NY; 33 Wake Forest Baptist Medical Center, Winston-Salem, NC; 34 Hadassah Medical Center, Jerusalem, Israel; 35 University of Chicago, Chicago, IL; 36 London Health Sciences Centre, London, Ontario, Canada; 37 University of Michigan Comprehensive Cancer Center, Ann Arbor, MI; 38 Dent Neurosciences Research Center Incorporated, Amherst, NY; 39 University of Rochester Medical Center, Rochester, NY; 40 Stanford Advanced Medical Center, Stanford, CA; 41 Ottawa Hospital, Ottawa, Ontario, Canada; 42 Columbia University Medical Center, New York, NY; 43 University of Texas Southwestern Medical Center Dallas, TX; 44 Kaiser Permanente—Redwood City Medical Cente, Redwood City, CA; 45 Colorado Neurological Institute, Englewood, CO; 46 Sunnybrook Health Science Centre, Toronto, Ontario, Canada; 47 HealthPartners Riverside Clinic, Minneapolis, MN; 48 University of California San Diego, San Diego, CA; 49 University of Wisconsin, Madison, WI; 50 Dartmouth Hitchcock Medical Center, Lebanon, NH; 51 University of Kansas Medical Center, Kansas City, KS; 52 University of Virginia, Charlottesville, VA; 53 University of Florida, Gainsville, FL; 54 Chaim Sheba Medical Center, Tel Hashomer, Israel; 55 Rambam Health Corporation, Haifa, Israel; 56 Henry Ford Health System, Detroit, MI; 57 Dana Farber Cancer Institute, Boston, MA; 58 Rabin Medical Center, Petach Tikvah, Israel

**Keywords:** anti-angiogenesis, gene therapy, glioblastoma, VB-111, viral immuno-oncology

## Abstract

**Background:**

Ofranergene obadenovec (VB-111) is an anticancer viral therapy that demonstrated in a phase II study a survival benefit for patients with recurrent glioblastoma (rGBM) who were primed with VB-111 monotherapy that was continued after progression with concomitant bevacizumab.

**Methods:**

This pivotal phase III randomized, controlled trial compared the efficacy and safety of upfront combination of VB-111 and bevacizumab versus bevacizumab monotherapy. Patients were randomized 1:1 to receive VB-111 10^13^ viral particles every 8 weeks in combination with bevacizumab 10 mg/kg every 2 weeks (combination arm) or bevacizumab monotherapy (control arm). The primary endpoint was overall survival (OS), and secondary endpoints were objective response rate (ORR) by Response Assessment in Neuro-Oncology (RANO) criteria and progression-free survival (PFS).

**Results:**

Enrolled were 256 patients at 57 sites. Median exposure to VB-111 was 4 months. The study did not meet its primary or secondary goals. Median OS was 6.8 versus 7.9 months in the combination versus control arm (hazard ratio, 1.20; 95% CI: 0.91–1.59; *P* = 0.19) and ORR was 27.3% versus 21.9% (*P* = 0.26). A higher rate of grades 3–5 adverse events was reported in the combination arm (67% vs 40%), mainly attributed to a higher rate of CNS and flu-like/fever events. Trends for improved survival with combination treatment were seen in the subgroup of patients with smaller tumors and in patients who had a posttreatment febrile reaction.

**Conclusions:**

In this study, upfront concomitant administration of VB-111 and bevacizumab failed to improve outcomes in rGBM. Change of treatment regimen, with the lack of VB-111 monotherapy priming, may explain the differences from the favorable phase II results.

**Clinical trials registration:**

NCT02511405

**Key Points**

1. GLOBE results did not reproduce the promising outcomes that were seen in the phase II study, in which patients were initially primed with VB-111 monotherapy.
2. Clinical, molecular and MRI data indicate that co-administration of VB-111 and bevacizumab blocked the VB-111 antitumor effect.
3. A randomized, placebo controlled, phase II study of neoadjuvant and adjuvant VB-111 for treatment of rGBM will soon open, applying important lessons from GLOBE.

**Importance of the Study**

Patients with glioblastoma have a median survival following diagnosis of only 1–2 years, and a high unmet need for effective therapies. Bevacizumab, the US standard of care for rGBM, has demonstrated a PFS benefit, but has not shown an advantage in OS. In a phase II study, VB-111, an anticancer gene therapy operating via vascular disruption/anti-angiogenesis and induction of a tumor directed immune response, showed a survival benefit with almost doubling the survival of patients with rGBM compared with literature reports of bevacizumab monotherapy. These results led to the design of the GLOBE study, a randomized, controlled, phase III, single registration study to assess the efficacy and safety of VB-111 combined with bevacizumab compared with bevacizumab monotherapy in patients with rGBM.

Glioblastoma (GBM) is an aggressive and devastating primary brain cancer accounting for approximately 13 000 new patients each year in the United States and approximately 240 000 new patients worldwide each year.^1^ With a median overall survival (OS) of less than 2 years, there is no known cause and no early detection in GBM. Research into the tumor microenvironment, biological preclinical studies, and comprehensive molecular characterization of GBM has led to many rational clinical investigations targeting the cancer cell or its microenvironment using small molecules, cytotoxic agents, radiation sensitizers, antibodies, with or without drug conjugates, peptides, cell base therapies, and viruses to name a few. Unfortunately, the summation of these results over these last 40 years has only shown clinically meaningful activity with radiation therapy, 2 chemotherapies (temozolomide, carmustine), 1 antibody (bevacizumab), and 1 device (Optune). Median OS with standard of care surgery, radiation therapy, and temozolomide is only 14.6 months.^2^ It is further sobering that a survival benefit has only been established in the frontline setting with radiation and chemotherapy, with or without a device. There is no established survival benefit in recurrent glioblastoma (rGBM) with any therapy to date, with median OS estimated as 24–44 weeks.^3–5^

Angiogenesis is essential for progression from low-grade to high-grade gliomas, and there is a clear correlation between degree of vascularization and increased malignancy.^6–8^ Once the tumor has its own vasculature, which often is leaky and inefficient,^9–11^ it proliferates at much higher rates.^12^

High tumor vascularity resulting from elevated production of pro-angiogenic growth factors, including vascular epithelial growth factor (VEGF),^13,14^ has led to the development of therapies targeting pro-angiogenic signaling pathways.^15^ Anti-angiogenic therapies that specifically target VEGF and its receptors fall into 2 general categories: antibodies or tyrosine kinase inhibitors.^16^ Bevacizumab, a humanized monoclonal antibody for VEGF-A, was approved for use in rGBM in 2009 after it was shown to improve progression-free survival (PFS)^17,18^; however, despite promising initial data and widespread exploration of anti-VEGF therapies in rGBM, randomized phase II trials have not demonstrated an OS benefit for patients with rGBM.

VB-111 is an anticancer gene therapy with a dual mechanism of action: (i) vascular disruption/anti-angiogenesis leading to tumor starvation and (ii) induction of a tumor directed immune response (Fig. 1). VB-111 is based on non-integrating, replication-deficient adenovirus type 5 vector, which carries a transgene for a chimeric death receptor that connects intracellular Fas to human tumor necrosis factor (TNF) receptor 1. Binding of TNFα to the chimeric receptor activates the Fas pro-apoptotic pathway and leads to a vascular-targeted anti-angiogenic effect. The activity of the transgene is specifically restricted to tissues that endogenously activate the semi-artificial pre-proendothelin 1 (PPE-1)–3x promoter, namely, angiogenic endothelial cells.^19–22^ VB-111 also promotes specific intratumor activation of the immune system, seen by an increase in tumor-infiltrating CD8 cells, thereby inducing an antitumor immune response such as seen in viral immuno-oncology.^23,24^

**Fig. 1:**
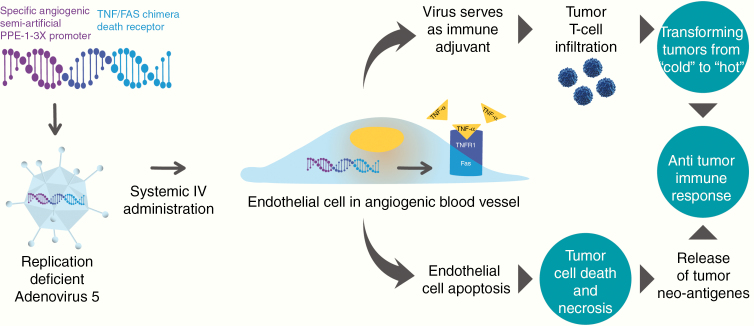
VB-111 dual mechanism of action: (1) Targeting tumor vasculature by apoptosis of angiogenic endothelial cells. (2) Induction of an antitumor immune response.

The safety and tolerability of VB-111 were assessed in 4 phase I/II clinical trials. The drug was proven to be safe and well tolerated in patients with advanced metastatic cancer at doses of up to 1 × 10^13^ viral particles (VPs),^23,25,26^ including in patients with rGBM, when given in combination with bevacizumab. NCT01260506, a phase I/II study of VB-111 rGBM, demonstrated a significant survival benefit for patients treated with VB-111 monotherapy that was continued upon progression with combination treatment of VB-111 and bevacizumab (VB-111 “primed combination”), compared with patients treated with limited exposure (LE) to VB-111. Median PFS was 90 versus 60 days for the primed combination vs LE group (hazard ratio [HR], 0.36; 95% CI: 0.14–0.93; *P* = 0.032), and median OS was 414 days versus 223 days (HR, 0.48; 95% CI: 0.23–0.99; *P* = 0.043). A survival advantage was also seen in comparison to literature reports of 694 rGBM patients treated with bevacizumab monotherapy across 8 studies. The 12-month OS in the primed combination group (57%) was double that of the historical controls (24%) (*P* = 0.03).^27^ Following these results, a pivotal, randomized, controlled study was designed to test whether upfront combination therapy with VB-111 and bevacizumab in patients with rGBM is associated with survival benefit compared with bevacizumab monotherapy.

## Materials and Methods

### Study Objectives

The objectives of the GLOBE study, NCT02511405, were to determine the safety and efficacy of combination treatment of VB-111 and bevacizumab compared with bevacizumab monotherapy in patients with rGBM. The primary endpoint was OS, and secondary endpoints were objective response rate (ORR) using Response Assessment in Neuro-Oncology (RANO) criteria and PFS.

### Patient Eligibility

Eligible participants were adults aged >18 years with first or second progression of histologically confirmed rGBM, who had received previous treatment with standard of care radiotherapy and temozolomide. Additional key inclusion criteria included KPS of at least 70%, life expectancy of at least 3 months, an interval of at least 12 weeks since the cessation of radiotherapy, and measurable disease by RANO criteria^28^ at time of progression. Patients treated with steroids had to be on a stable or decreasing dose.

Exclusion criteria included prior anti-angiogenic therapy, history of recent grade 2 or higher CNS hemorrhage, gastrointestinal bleeding or pulmonary hemorrhage/hemoptysis, inherited bleeding diathesis or significant coagulopathy at risk of bleeding, surgical treatment or significant trauma within 4 weeks, active vascular disease, proliferative and/or vascular retinopathy, inadequately controlled hypertension, history of gastrointestinal perforation or abscess.

### Study Design

This was a phase III multisite, international, randomized, open-label, controlled trial. Study design and treatment regimens were determined in agreement with an FDA special protocol assessment. Eligible patients with rGBM were randomized 1:1 to receive either VB-111 at 1 × 10^13^ VPs every 8 weeks in combination with bevacizumab 10 mg/kg every 2 weeks (combination arm) or bevacizumab monotherapy 10 mg/kg every 2 weeks (control arm). Treatment assignment was determined by central randomization and was stratified by age, KPS, and first or second progression. Disease characteristics, including local assessment of the prognostic factors O^6^-methylguanine-DNA methyltransferase (MGMT) methylation, epidermal growth factor receptor variant III (EGFRvIII), and isocitrate dehydrogenase 1 (IDH1) mutation, were collected from patients’ medical history (if available). Primary endpoint was OS, defined as the time from randomization until death from any cause.

Upon evidence of progressive disease by RANO (defined as ≥25% increase in the sum of enhancing lesion diameters), continuation or discontinuation of study therapy was decided per physician’s discretion, as long as the patient did not have increase in tumor measurements >50% or any confirmed T2/fluid attenuated inversion recovery and/or clinical deterioration. All patients who discontinued study drug were treated according to standard of care, and there was no crossover from bevacizumab monotherapy to VB-111. All efforts were made to collect post-study MRIs, health related quality of life measures, follow-up of anticancer treatments, and survival data every 2–3 months until the patient expired. Dose reductions of VB-111 and bevacizumab were not allowed. Repeat VB-111 dosing was delayed for patients who experienced a drug-related adverse event (AE), until the severity of the event was no more than Common Terminology Criteria for Adverse Events (CTCAE) grade 1. All patients received concomitant pre-dose acetaminophen to mitigate posttreatment fever, and pre-dose dexamethasone (10 mg) followed by 4 mg twice daily for 3 days post dosing to prevent potential cerebral edema.

### MRI and Radiographic Response Evaluation

Contrast and non-contrast brain MRIs were collected every 8 weeks and assessed both locally and by a central blinded independent radiology review (VirtualScopics, Rochester, New York). This report presents only the central assessments.

Secondary endpoints of response and progression were assessed using the international criteria proposed by the standard RANO working group.^28^ Best tumor response was assessed using RANO criteria, and PFS was measured from randomization to the date of progression or death. If neither of these occurred, PFS was censored at the date of last dose, date of initiation of alternative anticancer therapy, or date of last radiologic assessment, whichever was later.

Additional post-hoc quantitative tumor volumetric analysis was performed by a separate laboratory using contrast-enhanced T1-weighted digital subtraction maps and segmentation techniques described previously.^29–34^ Volumetric equivalent definitions for progression (>40% increase) and response (>65% reduction) were used to interpret quantitative tumor volumetric analyses per the modified RANO criteria. This analysis included comparison of the MR images with those of patients from the phase II study.

### Safety

Adverse events were recorded from the time of consent until 2 months after the last infusion and were assessed for seriousness, relatedness to study drug, and severity according to CTCAE version 4.0. Safety laboratory (blood hematology, chemistry, and urinalysis) was analyzed by a central laboratory. Vital signs, physical examination, and ECG were assessed.

### Study Oversight

The study was conducted in accordance with the Declaration of Helsinki and International Conference on Harmonization Guidelines for Good Clinical Practice. Study protocol was written by members of the trial management committee and was approved by the relevant institutional review boards of the participating sites. All participants provided written informed consent before commencement of study procedures. An independent Data and Safety Monitoring Committee met on regular intervals to monitor the safety and efficacy data as they accumulated.

### Statistical Analysis

All patients meeting the eligibility criteria who signed a consent form and were randomized were evaluated for efficacy. Safety was evaluated in participants who received at least one dose of study medication. The trial was planned to enroll 252 patients, randomized at a 1:1 ratio to either VB-111 with bevacizumab or bevacizumab alone. The sample size calculation assumptions were based on data from the control arm of the bevacizumab plus irinotecan study included in Genentech’s briefing document to the FDA (12-mo survival of 37.6%). The 12-month survival rate of VB-111 combined with bevacizumab was assumed to be 55% based on data from the phase II study VB-111–122, where patients were treated with VB-111 monotherapy, followed at progression by bevacizumab. It was expected that the survival following treatment with VB-111 combined with bevacizumab would be no worse than VB-111 monotherapy. A sample size calculation of 126 patients per arm provided statistical power of 89% for finding a difference significant at the 5% level, assuming a proportional hazards model, with an HR of 0.611 (=ln (.55)/ln (.376)).

Final analysis was performed after 189 patients had died. The final analysis compared the survival curves of the 2 treatment arms using the Cox regression model with the stratification variables of age (≤60 y vs >60 y) KPS (<80 vs ≥80), and first versus second progression, as adjusting covariates. Continuous variables (eg, reductions in tumor volume) were compared between the 2 treatment groups using a *t*-test. Proportions were compared using chi-squared tests.

Predefined subgroup analysis was performed for the primary and secondary efficacy endpoints, for parameters including, KPS, first or second disease progression, tumor volume at study enrollment, MGMT methylation status, sex, age, and country. Additional post-hoc subgroup analyses assessed the association between posttreatment fever and survival. A comparison was done between the baseline characteristics of the patients in the phase II and phase III studies and their correlation to the efficacy results in each study.

Analysis of the quantitative MRI data assessed the correlation between initial percentage change in tumor volume and OS by linear regression. *P*-values represent the level of significance based on whether the slope of the best fit line deviates from zero (GraphPad Prism v7.0e). Initial tumor volumetric response in the phase II and III studies was compared by ANOVA and Tukey’s multiple comparisons test.

## Results

### Patient Baseline Characteristics

Between August 2015 and January 2017, two hundred fifty-six patients were enrolled at 57 sites in the US, Canada, and Israel. Patient characteristics are summarized in Table 1. The mean age of patients was 55 years, 67% were male, 91% Caucasian, and 21% had a KPS lower than 80. Median time since initial diagnosis was 11.5 months, and 74% were at their first progression. Median baseline tumor area (product of the perpendicular dimensions of the tumor) was 1334 mm^2^ and 1190 mm^2^ in the combination and control arms, respectively. The treatment arms were well matched for baseline demographic and disease characteristics, including distribution of the prognostic factors MGMT methylation, EGFRvIII, and IDH mutations.

**Table 1:** Baseline patient characteristics

Characteristics	VB-111 + Bevacizumab, *n* (%)	Bevacizumab, *n* (%)
	(N = 128)	(N = 128)
Mean age, y (SD)	55.4 (11.20)	54.5 (12.21)
Age group		
≤60	82 (64.1)	83 (64.8)
>60	46 (35.9)	45 (35.2)
Sex		
Male	82 (64.1)	89 (69.5)
Female	46 (35.9)	39 (30.5)
Race		
White	117 (91.4)	117 (91.4)
Black/African American	1 (0.8)	3 (2.3)
Asian	3 (2.3)	2 (1.6)
American Indian/Alaska Native	0	0
Native Hawaiian/Pacific	0	1 (0.8)
Other	4 (3.1)	4 (3.1)
Missing	3 (2.3)	1 (0.8)
MGMT methylation status		
Yes	20 (15.6)	26 (20.3)
No	50 (39.1)	52 (40.6)
Not determined	35 (27.3)	27 (21.1)
Missing	23 (18.0)	23 (18.0)
EGFRvIII mutant		
Yes	26 (20.3)	24 (18.8)
No	27 (21.1)	24 (18.8)
Not evaluable	41 (32.0)	49 (38.3)
Missing	34 (26.6)	31 (24.2)
IDH1 mutation		
Mutated	13 (10.2)	12 (9.4)
Unmutated	78 (60.9)	84 (65.6)
Missing	37 (28.9)	32 (25.0)
Baseline KPS		
70	33 (25.8)	20 (15.6)
80	39 (30.5)	42 (32.8)
90	39 (30.5)	30 (23.4)
100	15 (11.7)	15 (11.7)
missing	2 (1.6)	21 (16.4)
Median time, mo, since initial diagnosis (range)	12.36 (2.3, 140.0)	11.09 (2.0, 70.0)
Disease classification		
Glioblastoma	117 (91.4)	125 (97.7)
Gliosarcoma	11 (8.6)	3 (2.3)
Progression		
First	92 (71.9)	97 (75.8)
Second	36 (28.1)	31 (24.2)
Tumor area (mm^2^), median sum of product of diameters of all target lesions (range)	1334.5 (186, 6212)	1190.2 (165, 8661)
Tumor volume, mean	23.9 cc (23 931 uL)	27.3 cc (27 265 uL)

### Disposition and Study Treatment

**Fig. 2:**
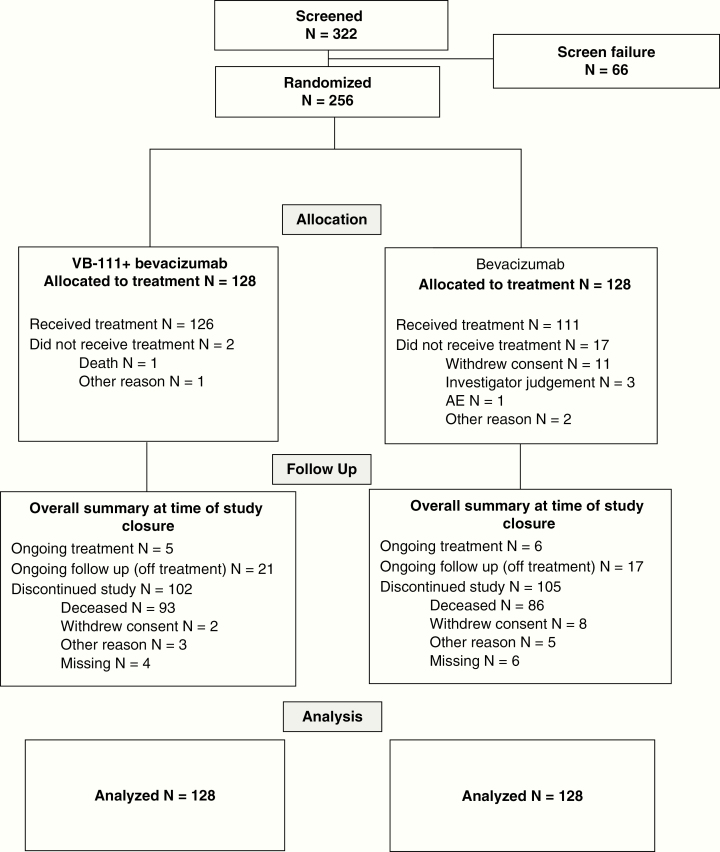
Study disposition CONSORT diagram.

All 256 patients enrolled and randomized to the trial (128 per arm) were included in the efficacy analysis (intent to treat) (Fig. 2). In total, 237 patients received at least one dose of study treatment and were included in the safety analysis set. Nineteen patients (7.4%) discontinued before receiving any study treatment (2 in the combination arm and 17 in the control arm). Fifty-five patients (21.5%) discontinued study treatment after receiving at least one treatment dose. The most frequent reasons for treatment discontinuation were disease progression (7 [5.5%] and 13 [10.2%] patients in the combination and control arms, respectively), followed by AEs (5 [3.9%] patients each in the arms) and withdrawal of consent (7 [5.5%] and 2 [1.6%] patients in the combination and control arms).

At the time of study closure, on November 3, 2017, eleven patients (4.3%) were continuing study treatment, 38 (14.8%) were on follow-up without study treatment, and 207 (80.9%) discontinued the study. The most common reason for study discontinuation was patient death (179 patients [69.9%], with 93 [72.7%] deaths in the combination arm, and 86 [67.2%] in the control arm). Median exposure to VB-111 was 4 months (2 infusions, range 1–8 infusions). Median exposure to bevacizumab was 3.59 months (7 infusions) in the combination arm versus 4.0 months (8 infusions) in the control arm.

### Efficacy

**Fig. 3:**
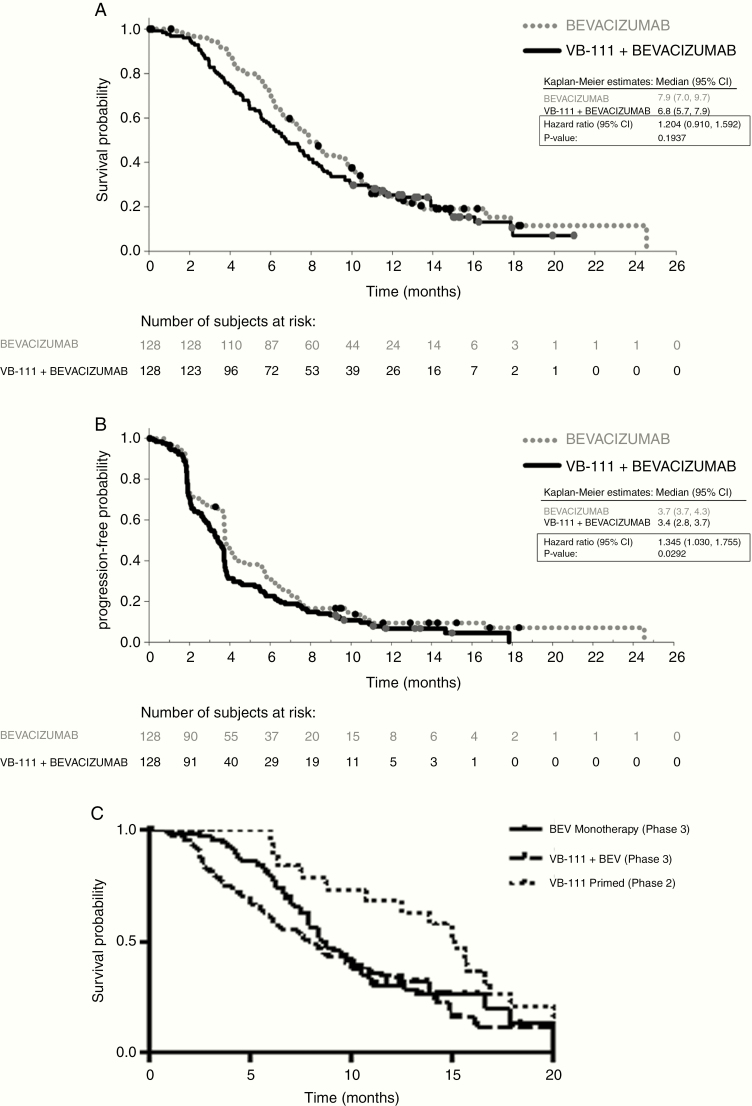
Efficacy endpoints Kaplan–Meier curves. (A) OS; (B) PFS; (C) OS of patients with tumors <25 mL in phase II primed combination, GLOBE unprimed combination, and bevacizumab control

The primary and secondary outcome goals in this study were not met (Fig. 3). Median OS for the intent-to-treat population was 6.8 months (95% CI: 5.7–7.9) in the combination arm versus 7.9 months (95% CI: 7.0–9.7) in the control arm. No statistically significant difference was observed in the OS time distributions between the 2 arms (HR, 1.204 [0.91, 1.59]; *P* = 0.19). The OS probabilities at 12 months were similar in the combination arm and control arm, 25.3% vs. 24.9%, respectively. Median PFS time was 3.4 versus 3.7 months in the combination versus control arms (HR, 1.30; 95% CI: 1.03–1.75; *P* = 0.03), pointing to ~1 week difference between the groups. ORR (complete or partial tumor response) was 27.3% in the combination arm versus 21.9% in the control arm (*P* = 0.30) with median duration of response of 3.7 versus 2.2 months. Of note, 7 patients in the combination arm (5.5%) achieved a complete response versus 2 patients in the control arm. OS among the responders was 11 months (combination) versus 8.5 months (control).

Quantitative MRI analyses revealed that both treatment arms had a similar tumor volumetric response (*P* = 0.92). The median percentage decrease in tumor volume was 58.3% in the combination group and 56.8% in the bevacizumab control. Interestingly, only in the patients in the combination arm was a significant negative linear correlation observed between initial percentage change in tumor volume and OS (*R*^2^ = 0.11, *P* = 0.0005), suggesting that the larger the decrease in tumor volume, the longer the OS. This was not the case in the bevacizumab monotherapy group, which showed no association between the degree of tumor size reduction and OS (*R*^2^ = 0.001, *P* = 0.71) (Supplementary Figure 1A). Patients with a radiographic response using volumetric criteria (>65% reduction) had a significant survival difference compared with non-responders when controlling for age, baseline tumor volume, and treatment arm (HR, 0.58; *P* = 0.0014). This difference was statistically significant only for the responders in the combination arm (median OS time 9.3 mo vs 6.0 mo; HR, 0.52; *P* = 0.003).

Further analysis of interactions between treatment and prognostic factors identified that subgroups of patients who had a baseline tumor size smaller than 15 mm^3^ and patients with a single lesion had trends for better outcomes on combination treatment compared with control. In patients with baseline tumor size <15 mm^3^, OS in the combination arm was 9.2 versus 8.3 months with control (HR, 0.80; *P* = 0.32), and 12-month OS was 39% versus 27%. In the subgroup of patients with 1 lesion at baseline (defined by the central MRI reading center) the estimated HR for combination therapy versus control was 0.66 (*P* = 0.13). Patients in the combination arm with a posttreatment febrile reaction had an OS of 8.3 months compared with 5.5 months in patients with no fever, indicating that fever may be a biomarker for VB-111 efficacy. None of the subgroups defined by age, sex, country, MGMT methylation status, number of progressions, or KPS showed longer OS times with combination treatment.

### Post-Hoc Comparison to Phase II Results

The MRIs of 205 patients who had adequate follow-up MRI data to determine volumetric response were analyzed and compared with MRIs from the phase II study. Mean baseline tumor volume was not substantially different between the 2 GLOBE arms (27.3 cc in the bevacizumab group vs 23.9 cc in the combination group) and was slightly lower (20.3 cc) in the primed combination group of the phase II study. Continuous measures of baseline tumor volume were prognostic for OS time in all treatment groups when controlling for therapy and age (Cox, *P* < 0.001; HR, 1.02 per cc tumor volume).

In patients with tumors smaller than 25 mL, the phase II primed combination group had a significant OS advantage compared with both GLOBE arms: compared with the upfront combination arm (median OS times 7 mo vs 15 mo; HR, 0.53; *P* = 0.009) as well as compared with bevacizumab control (median OS 8.5 mo vs 15 mo; HR, 0.58; *P* = 0.025) (Figure 3C). In tumors larger than 25 mL, primed combination treatment did not have such a survival advantage.

A comparison of the initial tumor volumetric response in the phase II and III studies suggests that there was a significantly larger reduction in enhancing tumor volume in both phase III arms compared with VB-111 monotherapy priming in the phase II study (ANOVA, *P* = 0.0274; phase III bevacizumab vs phase II VB-111 monotherapy adjusted *P* = 0.0190, phase III combination arm vs phase II VB-111 monotherapy adjusted *P* = 0.0149) (Supplementary Figure 1B). This suggests that the typical initial volumetric change observed with VB-111 monotherapy is not repeated when VB-111 is administered with concomitant bevacizumab.

### Safety

Nearly all (98%) of the patients enrolled to the GLOBE study experienced at least one treatment emergent adverse event (TEAE). Since all patients were treated with bevacizumab either with or without concomitant VB-111, bevacizumab treatment has contributed to the AE occurrence. Study drug tolerability was similar between the 2 groups, evidenced by a similar rate of TEAE leading to treatment discontinuation in the combination and bevacizumab monotherapy arms; 18.3% and 17.1%, respectively. Tables 2 and 3 summarize study safety data and indicate a higher rate of serious AEs (SAEs) (51.6% vs 29.7%), and grades 3–4 AEs (67.5% vs 39.6%) in the combination arm compared with the bevacizumab control arm. This was mainly attributed to a higher rate of grade 3 fever events aligned with the known safety profile of viral therapies such as VB-111: 5 patients (4%) with combination compared with none with bevacizumab, and grades 3–4 CNS events commonly associated with GBM diagnosis reported by 34 patients (27%) in the combination arm compared with 16 patients (14.4%) receiving bevacizumab monotherapy. These AEs included seizures (9 vs 4), headache (5 vs 1), syncope (4 vs 0), and confusion (5 vs 0). Increased SAE rate was especially apparent in patients with large tumors.

**Table 2:** Treatment emergent adverse events

Patients with	VB-111 + Bevacizumab (*n* = 126)	Bevacizumab (*n* = 111)	Overall (*n* = 237)
	N (%)	N (%)	N (%)
Any AE	125 (99.2)	107 (96.4)	232 (97.9)
Any serious AE	65 (51.6)	33 (29.7)	98 (41.4)
Any VB-111 related AE	91 (72.2)	0	91 (38.4)
Any bevacizumab related AE	85 (67.5)	60 (54.1)	145 (61.2)
Any CTCAE grade 3–5 AE	85 (67.5)	44 (39.6)	129 (54.4)
Any AE leading to treatment discontinuation	23 (18.3)	19 (17.1)	42 (17.7)
Any AE leading to death*	6 (4.8)	2 (1.8)	8 (3.4)

* Per protocol events which are part of the natural course of the disease under study (ie, disease progression) should not have been reported as AEs and are not included in this row.

**Table 3:** Frequent TEAEs (reported by >10%) by preferred term

Preferred Term	VB-111 + Bevacizumab (*n* = 126)	Bevacizumab (*n* = 111)	Overall (*n* = 237)
	N (%)	N (%)	N (%)
Fatigue	41 (32.5)	30 (27.0)	71 (30.0)
Headache	37 (29.4)	26 (23.4)	63 (26.6)
Hypertension	24 (19.0)	27 (24.3)	51 (21.5)
Pyrexia	44 (34.9)*	4 (3.6)	48 (20.3)
Confusional state	26 (20.6)	13 (11.7)	39 (16.5)
Seizure	20 (15.9)	15 (13.5)	35 (14.8)
Hemiparesis	20 (15.9)	14 (12.6)	34 (14.3)
Nausea	19 (15.1)	14 (12.6)	33 (13.9)
Fall	17 (13.5)	14 (12.6)	31 (13.1)
Diarrhea	19 (15.1)	9 (8.1)	28 (11.8)
Muscular weakness	20 (15.9)	8 (7.2)	28 (11.8)
Dysphonia	17 (13.5)	10 (9.0)	27 (11.4)
Disease progression	17 (13.5)	9 (8.1)	26 (11.0)
Vomiting	21 (16.7)	5 (4.5)	26 (11.0)
Aphasia	18 (14.3)	7 (6.3)	25 (10.5)
Asthenia	14 (11.1)	9 (8.1)	23 (9.7)
Chills	22 (17.5) *	1 (0.9)	23 (9.7)
Gait disturbance	15 (11.9)	7 (6.3)	22 (9.3)
Insomnia	16 (12.7)	6 (5.4)	22 (9.3)
Decreased appetite	13 (10.3)	8 (7.2)	21 (8.9)
Urinary tract infection	14 (11.1)	4 (3.6)	18 (7.6)
Alanine aminotransferase increased	13 (10.3)	4 (3.6)	17 (7.2)
Influenza-like illness	13 (10.3)*	1 (0.9)	14 (5.9)

* AEs most frequently considered by investigators as related to VB-111.

The most common AEs, reported by >10% of the patients, included fatigue, headache, hypertension, pyrexia, confusion, seizures, hemiparesis, nausea and vomiting, fall, diarrhea, muscular weakness, dysphonia, disease progression, and aphasia. Infusional AEs (defined as AEs related to study medication occurring within 24 hours of infusion) were more frequent in the combination arm, 50/126 (39.7%), compared with the control arm, 16/111 (14.4%). Most of this difference was related to transient mild-moderate pyrexia, chills, fatigue, and influenza-like illness known to be associated with VB-111, being a viral-based therapy.

## Discussion

In the GLOBE trial, upfront combination of VB-111 and bevacizumab failed to increase OS and PFS. GLOBE results did not reproduce the promising outcomes that were seen in the phase II study, where patients initially treated with VB-111 monotherapy that was continued after disease progression in combination with bevacizumab had durable tumor growth attenuation and a median OS time of 414 days.^27^

Unfortunately, GLOBE adds to several recent phase III GBM studies that were negative despite promising results in the preceding phase II. The disappointing results often indicate that the studied drug is not efficacious in the studied indication; however, it is prudent to carefully assess any other factors that may have led to the conflicting results. Therefore, the differences between the phase II and III studies were closely inspected.

The distributions of prognostic factors were generally comparable between the 2 studies and could not explain the different survival outcomes. However, there was a major difference in the treatment regimen used in each of the studies. Thus, it is hypothesized that the contradictory outcomes are related to the lack of VB-111 monotherapy priming in the GLOBE study. This hypothesis is supported by the unfavorable survival results of the small group of patients in the phase II study’s unprimed combination group that received, just as in GLOBE, concomitant VB-111 and bevacizumab. Unfortunately, the results of the unprimed combination group were not available at the time of the design and conduct of GLOBE. Further support for this assumption is provided by preclinical studies in mice assessing tumor burden in the Lewis lung carcinoma model, where coadministration of bevacizumab and VB-111 blocked the antitumor effect of VB-111.^35^

Although VB-111 and bevacizumab are both anti-angiogenic agents, their mechanism of actions differ: bevacizumab antagonizes VEGF, while VB-111 directly disrupts the angiogenic vessels and induces a tumor directed immune response. It is plausible that the concomitant administration of bevacizumab interfered with VB-111’s action by several possible mechanisms. First, at the cellular level, bevacizumab normalizes angiogenic cells, which are the target for VB-111, therefore in the absence of angiogenic cells, VB-111 will not be able to trigger an effect. Second, at the molecular level, the PPE-1 promoter is activated by VEGF, and lack of VEGF reduces PPE-1–3x promoter-regulated transgene expression and prevents VB-111 activity.^21^ Third, at the tissue level, VB-111 acts by disrupting angiogenic blood vessels, which promotes tumor starvation but also enables immune cell penetration, while bevacizumab triggers the closure of the blood–brain barrier and may prevent VB-111-mediated recruitment of immune cells into the CNS. Fourth, being regulated by a tissue- and condition-specific promoter, VB-111 is not immediately active upon dosing. By the time of VB-111 activation, bevacizumab has already been administered, potentially blocking any further effect by VB-111.

The assumption that co-administration of VB-111 and bevacizumab blocked the VB-111 antitumor effect is supported by the unique MRI signature seen in the phase II trial after VB-111 monotherapy, which was not repeated in the GLOBE study, where both bevacizumab monotherapy and unprimed combination arms showed a similar volumetric response, typical for bevacizumab.

Although a similar tumor volumetric response was seen in both GLOBE treatment arms, only in the combination arm was the initial percentage change in tumor volume associated with increased OS, suggesting that tumor radiographic response with VB-111 is meaningful for survival. In the bevacizumab monotherapy arm, no correlation was seen between the degree of tumor size reduction and OS, resembling previous bevacizumab studies that have demonstrated improved PFS that is not associated with an OS benefit.

A recent study of neoadjuvant and adjuvant programmed cell death type 1 (PD-1) monoclonal antibody blockade in rGBM showed statistically significant improvements in OS and PFS with neoadjuvant treatment compared with the adjuvant treatment regimen.^36^ These results follow several negative phase III trials that have not demonstrated efficacy of adjuvant PD-1 blockade in GBM, and possibly point to a therapeutic window for efficacy of immunotherapy treatments in rGBM. This finding may be of relevance for VB-111, which has a dual mechanism of both anti-angiogenesis and immune stimulation via viral immune oncology, and warrants the assessment of VB-111 in the neoadjuvant setting. Indeed, a randomized, placebo controlled, phase II study of neo-adjuvant and adjuvant VB-111 for treatment of rGBM will soon open. Important lessons from GLOBE were applied to the study design and all patients will be primed with VB-111 monotherapy prior to any bevacizumab administration.

The identification of patient subgroups with trends for improved OS with combination treatment may be related to the drug’s mechanism of action. The improved outcomes associated with a post VB-111 febrile reaction are in accordance with similar observations in previous VB-111 studies and provide further support that fever is a potential biomarker for better survival with VB-111, secondary to the drug’s immunologic mechanism of action. The improved outcomes in the subset of patients with lower tumor volume at baseline indicate that large progressive rGBM tumors may have insufficient drug exposure.

Study drug tolerability was similar between the 2 treatment arms, yet a higher rate of SAEs and grades 3 and 4 AEs were reported with combination treatment. While it is not uncommon for combination treatments to be associated with an increased AE rate compared with monotherapies, it is also possible that the study’s open label design has contributed a reporting bias associated with increased AE reporting in the experimental treatment group. The increased AE rate observed in the combination arm was attributed to a higher rate of fever events, which is aligned with the known safety profile and the viral immuno-oncology properties of VB-111 and to higher rate of various CNS AEs such as seizures and confusion, related to the baseline malignancy. The increased AE rate was especially apparent among patients with a large tumor volume at baseline. Similar CNS toxicity is not expected to be prevalent upon administration of VB-111 in non-brain tumor indications.

In summary, although negative, we believe that the results of GLOBE may be due to the treatment regimen change and do not necessarily reflect the potential efficacy of VB-111 in different regimens. VB-111 is being further studied in GBM and other indications in primed treatment regimens.

## Supplementary Material

noz232_suppl_Supplementary_Figure_1

## Notes

Data presented within this article were previously presented in part at SNO 2018 and ASCO 2019.

## GLOBE Study Investigators

Avgeropoulos, Nicholas; University of Florida, Orlando Health, Orlando, FL

Beck, Joseph; Highlands Oncology Group, Rogers, Arizona

Benkers, Tara; Swedish Medical Center, Seattle, WA

Bokstein, Felix; Tel Aviv Sourasky Medical Center, Tel Aviv, Israel

Brenner, Andrew; University of Texas Health Science Center San Antonio, San Antonio, TX

Burton, Eric; University of Louisville Physician, Louisville, KY

Butowski, Nicholas; University of California, San Francisco, San Francisco, CA

Campian, Jian; Washington University School of Medicine, St. Louis, MO

Carrillo, Jose; University of California Irvine Medical Center, Orange, CA

Cloughesy, Timothy; University of California Los Angeles, Los Angeles, CA

de Groot, John; MD Anderson Cancer Center, Houston, TX

De Robles, Paula; Tom Baker Cancer Centre, Calgary, Alberta

Drappatz, Jan; Hillman Cancer Center, Pittsburgh, PA

Dunbar Irine; Piedmont Physicians Neuro-Oncology, Atlanta, Georgia

Fink, Karen; Neuro-Oncology Associates, Baylor University, Dallas, TX

Groves, Morris; Texas Oncology-Austin Midtown, Austin, TX

Han, Xiaosi; University of Alabama at Birmingham, Birmingham, AL

Hormigo Adila; Derald H. Ruttenberg Treatment Center, New York, NY

Jensen, Randy; Huntsman Cancer Institute at The University of Utah, Salt Lake City, UT

Kowalska, Agnieszka; Neurology Associates of Stony Brook, Stoney Brook, NY

Kumthekar, Pyriya; Northwestern University, Chicago, IL

Lee, Mijung; SUNY Upstate Medical Center, Syracuse, NY

Lesser, Glenn; Wake Forest Baptist Medical Center, Winston-Salem, NC

Lossos, Alexander; Hadassah Medical Center, Jerusalem, Israel

Lukas, Rimas; University of Chicago, Chicago, IL

Macdonald, David; London Health Sciences Centre, London, Ontario, Canada

Mammoser, Aaron; University of Michigan Comprehensive Cancer Center, Ann Arbor, MI

Mechtler, Laszlo; Dent Neurosciences Research Center Incorporated, Amherst, NY

Mohile, Nimish; University of Rochester Medical Center, Rochester, NY

Nagpal, Seema; Stanford Advanced Medical Center, Stanford, CA

Nicholas, Garth; Ottawa Hospital, Ottawa, Ontario, Canada

Kreisl, Teri; Columbia University Medical Center, New York, NY

Pan, Edward; University of Texas Southwestern Medical Center; Dallas, TX

Peak, Scott; Kaiser Permanente—Redwood City Medical Cente, Redwood City, CA

Pearlman, Michael; Colorado Neurological Institute, Englewood, CO

Perry, James; Sunnybrook Health Science Centre, Toronto, Ontario, Canada

Peterson, Richard; HealthPartners Riverside Clinic, Minneapolis, MN

Piccioni, David; University of California San Diego, San Diego, CA

Robins, Henry; University of Wisconsin, Madison, WI

Ronan, Lara; Dartmouth Hitchcock Medical Center, Lebanon, NH

Salacz, Michael; University of Kansas Medical Center, Kansas City, KS

Schiff, David; University of Virginia, Charlottesville, VA

Tran, David; University of Florida, Gainsville, FL

Zach, Leor; Chaim Sheba Medical Center, Tel Hashomer, Israel

Tzuk-Shina, Tzahala; Rambam Health Corporation, Haifa, Israel

Walbert, Tobias; Henry Ford Health System, Detroit, MI

Wen, Patrick; Dana Farber Cancer Institute, Boston, MA

Youst, Shlomit; Rabin Medical Center, Petach Tikvah, Israel

**Conflict of interest statement.** YC, TRM, SFS, and NL-S are employees of VBL Therapeutics. LSF is a statistical consultant to VBL Therapeutics and his institute (Gertner Institute for Epidemiology and Health Policy Research) received payment for the statistical advice and analysis provided. TFC is a paid consultant for VBL, Trizel, Medscape, Bayer, Amgen, Odonate Therapeutics, Pascal Biosciences, Bayer, Del Mar Pharmaceuticals, Tocagen, Karyopharm, GW Pharma, Kiyatec, Abbvie, Boehinger Ingelheim, VBI, Deciphera, Agios, Merck, Roche, Genocea, Celgene, Puma, Lilly, BMS, Cortice, Wellcome Trust, Novocure, Novogen, Boston Biomedical, Sunovion, Human Longevity, Insys, ProNai, Pfizer, Notable labs, and Medqia; stock options for Notable Labs; board member for 501c3 Global Coalition for Adaptive Research and has a provisional patent on compositions and methods for treating cancer with UCLA. AB has no conflicts. JFG has grant and research support through Grant or Research Support: Sanofi-Aventis, Astrazeneca, EMD-Serono, Eli Lilly, Novartis, Deciphera Pharmaceuticals, and Mundipharma; paid consultant for Celldex, Deciphera Pharmaceuticals, AbbVie, FivePrime Therapeutics, Inc., GW Pharma, Carthera, Eli Lilly, Kadmon, Boston Biomedical Inc., Taiho Pharmaceuticals, Kairos Venture Investments, Syneos Health, Monteris, Agios, Mundipharma Research, GenomiCare, Blue Earth Diagnostics, Del Mar Pharmaceuticals, Insightec, E-Health Now; advisory boards for Genentech, Celldex, Foundation Medicine, Inc., Novogen, Deciphera, Astrazeneca, Insys Therapeutics, Merck, Eli Lilly, Novella Clinical, Kiyatec, Blue Earth Diagnostics, Vanquish Oncology, Orsenix, Insightec, Prelude Therapeutics; stock ownership in Ziopharm Oncology, and Gilead; company employment with Ziopharm Oncology (spouse); other financial interests include DSMB memebership for VBL Therapeutics, Novella, and VBI Vaccines, Inc. NAB is a paid consultant for VBL Bayer, Amgen, Del Mar Pharmaceuticals, Tocagen, GW Pharma, Kiyatec, Abbvie, Boehinger Ingelheim, Deciphera, Merck, Roche, Lilly, BMS, Cortice, Wellcome Trust, Novocure, Boston Biomedical, Insys. LSF has no conflicts. JLC paid consultant for Alexion, Abbvie, Arbor Pharmaceuticals, Dova Pharmaceuticals, Inovio Pharmaceuticals, Incyte, Novocure. BME paid consultant for MedQIA, Agios Pharmaceuticals, Siemens Healthcare, Janssen Pharmaceuticals, Medicenna, Imaging Endpoints, Genentech, Novogen, Northwest Biopharmaceuticals, Image Analysis Group, Oncoceutics, BeiGene, and VBL. PYW has research support from Agios, Astra Zeneca, Beigene, Eli Lily, Genentech/Roche, Karyopharm, Kazia, MediciNova, Merck, Novartis, Oncoceutics, Sanofi-Aventis, VBI Vaccines; consulting with Agios, Bayer, Astra Zeneca, Blue Earth Diagnostics, Eli Lilly, Genentech/Roche, Immunomic Therapeutics, Karyopharm, Kiyatec, Puma, Vascular Biogenics, Taiho, Tocagen, Deciphera, VBI Vaccines; Speaker honorarium from Merck and Prime Oncology.

**Authorship statement.** Experimental design: YCC, NL-S, SFS, study implementation: TFC, AB, JFG, NAB, LZ, JLC, PYW, analysis and interpretation; TFC, YCC, TRM, NL-S, SFS, LSF, BE, AB, NAB, PYB. All authors were involved in manuscript writing, review and approval.
