# Supplementary figures and images for: A randomized controlled phase III study of VB-111 combined with bevacizumab vs bevacizumab monotherapy in patients with recurrent glioblastoma (GLOBE)
Source: Neuro Oncol. 2019 Dec 7;22(5):705–17. doi: 10.1093/neuonc/noz232 (PMC7229248; doi:10.1093/neuonc/noz232)

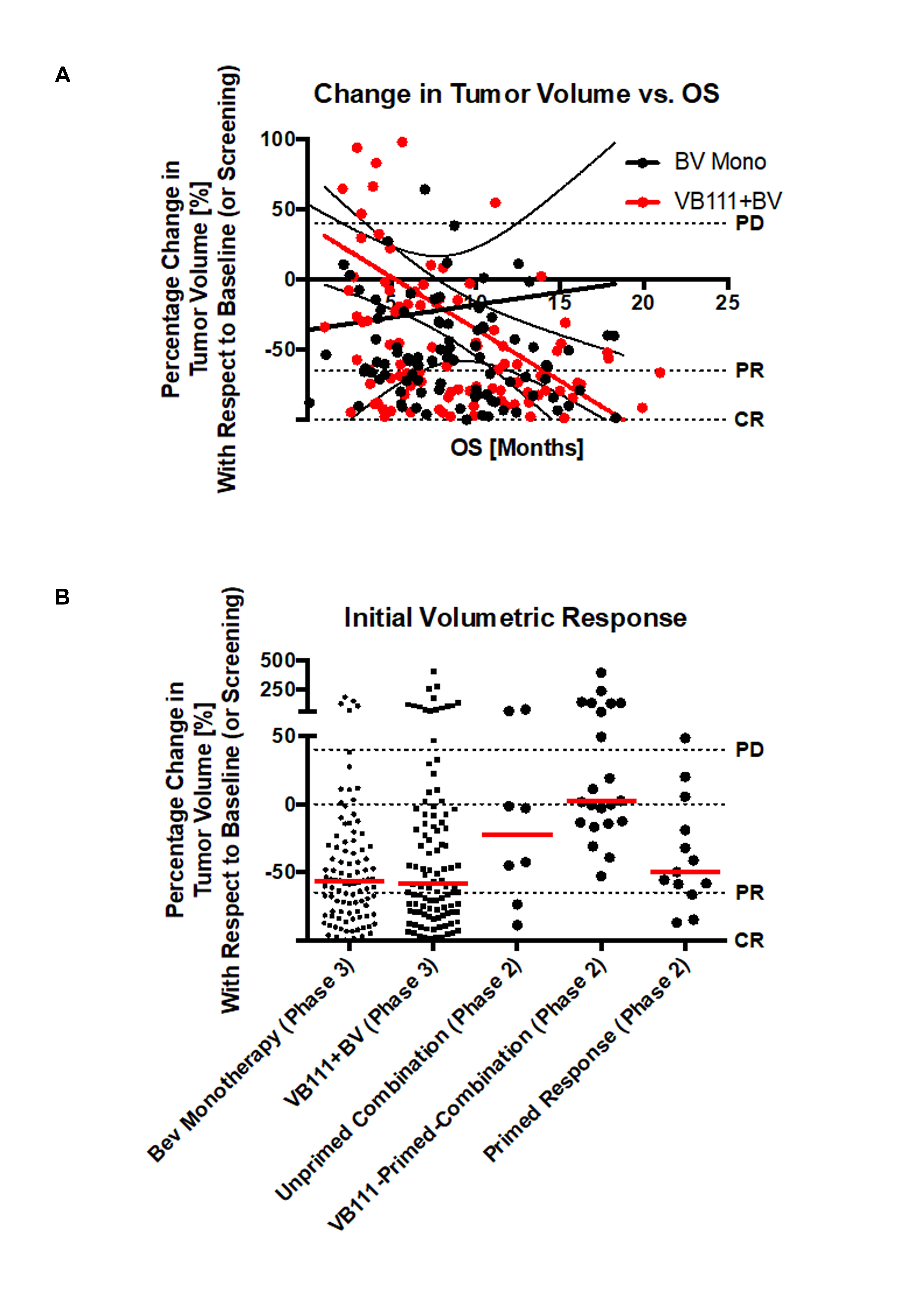

Supplement: noz232_suppl_Supplementary_Figure_1 [file noz232_suppl_supplementary_figure_1.png]
